# Subacute Hypophysitis with Panhypopituitarism as First Presentation of HIV and Syphilis Coinfection

**DOI:** 10.1155/2017/1489210

**Published:** 2017-04-16

**Authors:** Rute Alves, Margarida França

**Affiliations:** Clinical Immunology Unit, Centro Hospitalar do Porto, Porto, Portugal

## Abstract

Infection by* Treponema pallidum* still represents a clinical challenge due to its various forms of presentation. HIV coinfection added diversity and changed the natural history of syphilis as a systemic infection. We present a rare case of subacute hypophysitis and panhypopituitarism due to an early active neurosyphilis in a previously unknown HIV coinfected patient.

## 1. Introduction

Infection by* Treponema pallidum* is a common worldwide sexually transmitted disease that is a systemic condition, clinically polymorphic and typically evolving by stages. It is remarkable for its pathological interaction with human immunodeficiency virus (HIV): it facilitates HIV transmission and increases plasma and cerebrospinal fluid (CSF) HIV-RNA levels [1]. For its part, HIV itself can alter the clinical course of syphilis [2], adding semiologic diversity with more frequent early syphilitic neurological and ophthalmic involvement, increasing the probability of relapse, and making the diagnosis of neurosyphilis a clinical challenge.

Inflammatory condition of the pituitary gland (hypophysitis) is rare condition and its incidence (one case per 9 million people per year) has been estimated mainly from scarce case series reporting data from surgeries or necropsy specimens [3]. Secondary granulomatous form is even more unique (21 histologically confirmed cases) and syphilis is one of the documented etiologies [4]. Panhypopituitarism (hyposecretion of 3 or more of pituitary produced hormones) is one of the possible presentations. To the best of our knowledge, this is the second report in literature of symptomatic hypophysitis with panhypopituitarism due to acquired syphilis in an HIV patient.

## 2. Case Presentation

**Figure 1:**
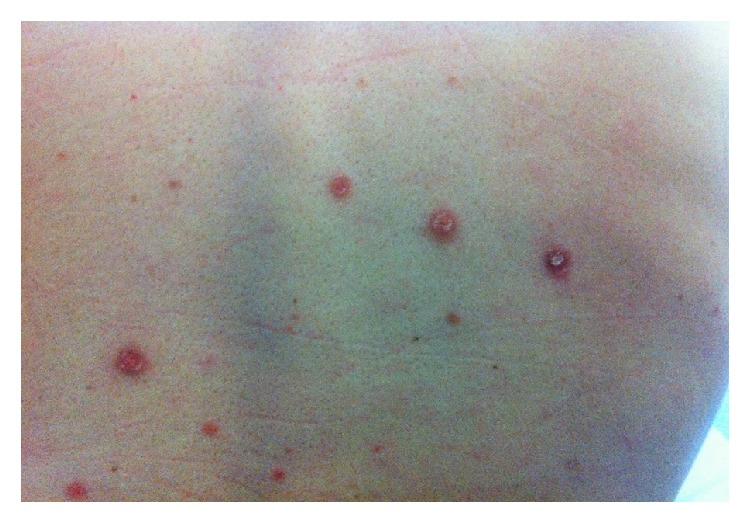
Generalized cutaneous erythematous maculae with distinct edges.

**Figure 2:**
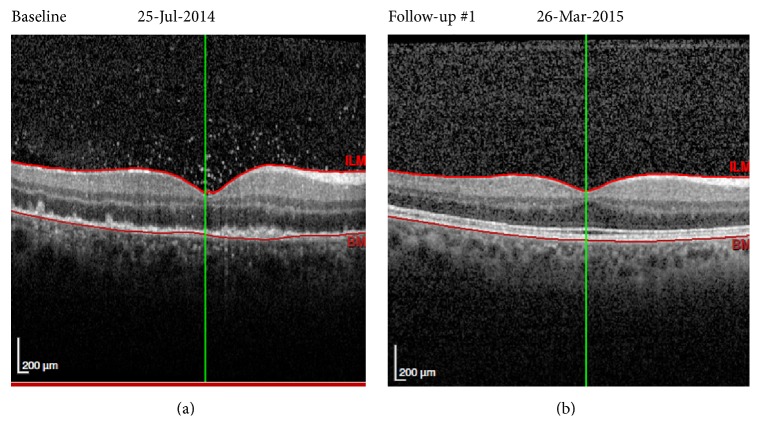
Macular optic computed tomography of right eye, during active disease (a). Small vitreous inflammatory agglomerates and inflammatory deposits in neurossensorial retina; diffuse macular thickening. After resolution (b).

In July 2014, a Portuguese 42-year-old male reported sudden painless right central loss of vision with no photophobia, discharge, or trauma. He had a history of malaise and mild and intermittent holocranial headaches, with no associated nausea, vomiting, or photophobia, for the last 3 months. The headaches became progressively severe in the last 15 days, interfering with sleep and daily activities, associated with progressively worsening gait imbalance and anorexia. He also noticed cold intolerance, erectile dysfunction, and decreased libido, without constipation, polydipsia, polyuria, or peripheral edema. He correlated the symptoms with exhausting working hours and he did not seek medical attention. He also referred to a generalized pruriginous skin rash with 1-month evolution. His past medical history was unremarkable, except for smoking habits and unprotected homosexual intercourse. At presentation in our emergency room he looked undernourished (BMI = 19 Kg/m^2^) with asymptomatic low blood pressure (90/62 mmHg). He had facial seborrheic eczema, generalized cutaneous erythematous maculae with distinct edges including palms and soles (Figure 1), a small nontender ulcer in the inferior lip, and a smooth nontender mild hepatomegaly. His pupils were equal, round, and reactive to light and accommodation and extraocular movements were intact, not suggestive of Argyll-Robertson pupils. He showed gait ataxia without proprioceptive impairment and bilateral positive finger-nose-finger test. He had a central scotoma in the right eye, with initially preserved peripheral vision that rapidly deteriorated; fundoscopy was highly suggestive of panuveitis, with fine keratic granulomatous precipitates, aqueous cells, flare grade 1, and choroidal and subretinal infiltration in the posterior pole (Figure 2(a)).

**Figure 3:**
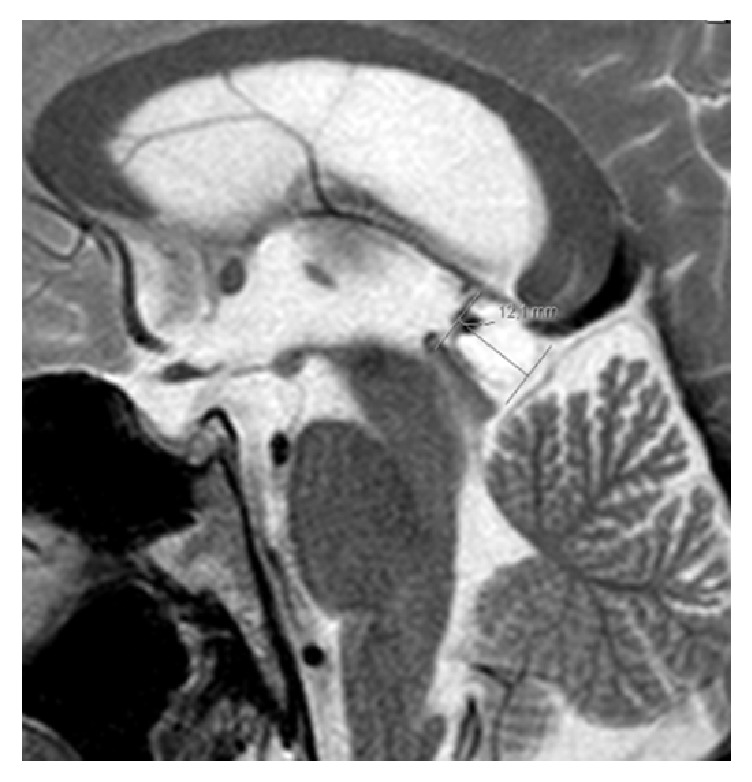
MRI of* sella turcica* at admission (August 2014): heterogeneous enlargement with peripheral contrast enhancement.

**Figure 4:**
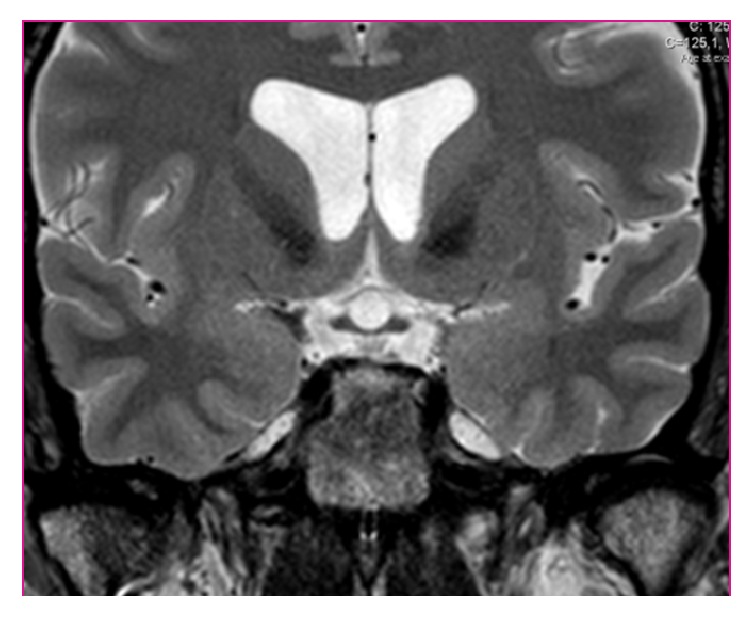
MRI of* sella turcica* at 10-month follow-up (May 2015): normal pituitary size with discrete heterogenous enhancement.

Cerebral computed tomography ruled out space occupying lesions or signs of high intracranial pressure. CSF analysis showed lymphocytic pleocytosis (48 cells/uL) with high level protein (0,89 g/L). Given the patient's social history with risk for sexually transmitted infections, HIV and syphilis with uveitis and meningitis were high on the differential. He was hospitalized for investigation. Initial laboratory results revealed leukopenia (3520/*μ*L) with lymphopenia (1340/*μ*L) and a positive HIV serology with CD4 cell count of 264/*μ*L (18,4%; CD4/CD8-ratio 0,34). HIV viral load was 54700 copies/mL (log⁡4,74). He had a serum VDRL positive at 64 “dils,” with positive IgM antibody. CSF VDRL was negative, and CSF HIV-RNA was 3290 copies/ml. Bacterial, fungal, viral, and mycobacterial organisms in CSF were excluded. Thyroid-stimulating hormone (TSH) was suppressed (0,26 mUI/mL) and free thyroxine (fT4) had a low value (0,43 ng/dl). MRI of the* sella turcica* showed pituitary heterogeneous enlargement with peripheral contrast enhancement, with no pituitary stalk thickening (Figure 3). Extended endocrine study also showed low levels of basal cortisol (1,5 *μ*g/dL), luteinising-hormone (0,6 mUI/ml), and testosterone (0,025 ng/mL). He underwent a 14-day regimen of penicillin G and was started on hormone reposition: high dose prednisolone (due to concomitant panuveitis) and levothyroxine (75 *μ*g qd). He was discharged after 15 days, with complete resolution of headache, improvement of visual acuity, and more coordinated and steady gait. Hormone values normalized within days. He was started on antiretroviral therapy in October 2014, with tenofovir/emtricitabine and efavirenz, presenting undetectable HIV-RNA since August 2015. In July 2016 he presented a CD4 cell count of 510/*μ*l (27,4%), positive TPHA, and negative (2 “dils”) VDRL. The fivefold decrease in VDRL titers (4 dils) occurred in the first four months after penicillin treatment. During follow-up hormone replacement therapies were gradually tapered along with neurological and ocular improvement until complete resolution (Figure 2(b)). MRI of the* sella turcica* 10 months later showed an almost normal pituitary size with discrete heterogenous enhancement (Figure 4). Nowadays the patient remains asymptomatic. In conclusion, HIV1 infection and an early syphilis infection (stage II) with neurological involvement—panuveitis, meningitis, and hypophysitis with panhypopituitarism—were diagnosed. The high activity of* T. pallidum* infection was illustrated by positive specific serum IgM antibody and the early neurosyphilis presentation with hypophysitis was corroborated by CSF abnormalities and MRI findings and sustained clinical, neurological, endocrinological, and serological recovery after specific treatment.

## 3. Discussion

Syphilis has been a persistent challenge for centuries and is now regaining attention along with the HIV pandemic [5]. The interaction between syphilis and HIV infection is complex and remains incompletely understood, despite the significative clinical experience with coinfected patients [6]. The course of syphilis within a depressed immune system clearly differs from that in HIV-negative patients [1, 2, 5], with accelerated development of early and late neurologic sequelae [7]. The diagnosis of neurosyphilis in coinfected patients requires a high index of suspicion, as the CSF abnormalities due to Treponema and HIV often overlap. On the other hand, CSF VDRL can be negative in the presence of active disease, and the favorable response to treatment plays a significant role to confirm the diagnosis. This case illustrates the multiplicity of attainments of syphilis, an infection that though recognized for centuries continues to cause surprise, especially in the HIV era.
